# Effects of Long Working Hours and Night Work on Subjective Well-Being Depending on Work Creativity and Task Variety, and Occupation: The Role of Working-Time Mismatch, Variability, Shift Work, and Autonomy

**DOI:** 10.3390/ijerph18126371

**Published:** 2021-06-12

**Authors:** Min-Gwan Shin, Yoon-Ji Kim, Tae-Kyoung Kim, Dongmug Kang

**Affiliations:** 1 Department of Medicine, Medical College, Pusan National University, Yangsan 50612, Korea; tlsalsrhks90@pusan.ac.kr; 2 Department of Preventive, and Occupational & Environmental Medicine, Medical College, Pusan National University, Yangsan 50612, Korea; harrypotter79@pusan.ac.kr; 3 Environmental Health Center of Asbestos, Pusan National University Yangsan Hospital, Yangsan 50612, Korea; 4 Medical Research Institute, Pusan National University, Yangsan 50612, Korea; 201882126@pusan.ac.kr; 5 Department of Occupational and Environmental Medicine, Pusan National University Yangsan Hospital, Yangsan 50612, Korea

**Keywords:** blue-collar worker, long working hours, night work, occupation, shift work, task characteristics, task variety, work creativity

## Abstract

This study explored the effects of long working hours (LW) and night work (NW) on subjective well-being and the modifying effects of work creativity and task variety (WCTV) and occupation. In addition, we examined the influence of working time-related variables including working-time mismatch, variability, shift work, and autonomy on the effects of LW and NW. This study used data from the 5th Korean Working Conditions Survey on 50,205 workers. LW and NW were defined as 52–60 h (L1) or >60 h (L2) per week, and 1–10 days (N1) or >10 days (N2) of night work per month. Multiple logistic regression was used to examine the effects of LW and NW and the modifying influences of WCTV and occupation. Differences in ORs of LW and NW caused by working time-related variables were investigated, to determine effect sizes and directions. A high level of WCTV alleviated the risks of LW and NW. White-collar workers were more vulnerable to the risk associated with NW. Regarding working-time related variables, working-time mismatch and variability increased the risks of LW and NW, respectively, while shift work alleviated the risks of NW. In countries where flexible work systems are not well utilized, working-time autonomy might not be associated with the risk of LW or NW. This study showed that it is necessary to comprehensively consider the occupation and task characteristics of individual workers performing LW or NW. Further studies of the modifying effects of working time-related variables on LW and NW are needed.

## 1. Introduction

In 2015, 16% of European workers worked more than 48 h per week and 19% worked nights [1]. These atypical work types have negative impacts on health such as on cardiovascular disease [2], metabolic disturbance [3,4], mortality [5,6], and depression [7]. However, although the effects of long working hours (LW) and night work (NW) have been investigated, few have addressed the synergistic effect of LW and NW using national-scale data.

Many studies have indicated that individual and environmental factors could influence the impact of LW and NW. For example, evening type workers showed high tolerance on NW [8,9] and female workers were more vulnerable to the risks posed by overtime work [10]. However, the effects of task characteristics, as compared with individual or environmental characteristics, on subjective well-being have received relatively little attention, though task characteristics such as complexity have been associated with exhaustion among night workers [11]. Furthermore, studies that examined the effect of task characteristics have reported conflicting results [12,13], which demonstrates the need to determine the extent to which task characteristics influence the effects of LW and NW on subjective well-being [14]. In addition, it is also possible to infer that the effects of LW and NW vary by occupation, even if workers had the same task characteristics, based on a previous study which showed that the risk of LW varied by occupation type (manual vs. non-manual) [15]. Therefore, it is necessary to examine the effects of LW and NW based on considerations of task characteristics and occupation type (blue collar vs. white collar).

A previous study reported that the effect of LW on life satisfaction depends on working-time mismatch [16]. Another study showed that the risk posed by LW was combined with working-time autonomy [17]. Because prior studies on the negative impacts of working-time mismatch and autonomy on subjective well-being were limited, there is a need to determine whether these working time-related variables increase or reduce the risks associated with LW and NW, and to what extent these risks depend on task characteristics and occupation. In addition, the studies to date have mostly highlighted the negative effect of shift work in terms of night shift or working-time variability [7,18]. However, night and shift work are technically different concepts [19], which should be examined separately.

Therefore, this study was conducted to examine the effects, including the synergistic effects, of LW and NW on subjective well-being, and to determine how these effects depend on occupation and task characteristics among Korean workers. In addition, we examined the modifying effects of working time-related variables including working-time mismatch, variability, shift work, and autonomy on the effects of LW and NW with respect to work creativity and task variety (WCTV) and occupation.

## 2. Materials and Methods

### 2.1. Participants

This study used data obtained from the 5th Korean Working Conditions Survey (KWCS) conducted in 2017. This survey was representative of the working environment in the Republic of Korea and included 50,205 workers the response rate was 0.449. A description of the sampling method used for the KWCS is described in the user guide report [20]. In KWCS, stratified random sampling was used to achieve nationwide representativeness. The present study included 50,205 economically active workers aged ≥15. Because participants did not respond to all questionnaire items, the numbers of samples included in the analyses differed slightly. The number of missing values for each variable among the 50,205 samples were as follows; (a) educational level (n = 52), (b) numbers of employee (n = 287), (c) subjective health conditions (n = 7), (d) monthly income (n = 4278), (e) working hours per week (n = 148), (f) WCTV (n = 156), (g) occupation (n = 93), (h) subjective well-being (n = 51), (i) working-time mismatch (n = 278), (j) working-time variability (n = 15), (k) shift work (n = 19), and (l) working-time autonomy (n = 111). All other variables had no missing values. All participants volunteered to participate in the KWCS and provided informed consent. Since KWCS derived data was public data from a national survey, IRB approval was not required.

### 2.2. Measurement

Subjective well-being was measured using the five-item World Health Organization Well-being Index. This index requires responses to five questions about overall feelings regarding cheerfulness, calmness, vigorousness, freshness, and interest over the previous two weeks [21]. Each response was scored using a six-point Likert scale ranging from 0 (none of the time) to 5 (all the time). The raw scores obtained (0 to 25 points) were transformed to scores from 0 (absence of well-being) to 100 points (maximal well-being) (Cronbach alpha = 0.925 in this study). The range 0 to 50 points was deemed low subjective well-being (LSW) and 51–100 as high. This dichotomization was performed for about 50 points because it has been previously used as a cut-off value for poor well-being [22]. WCTV variables were chosen to be in-line with the overview report of European Working Conditions Survey (EWCS) [1], and consisted of six components, that is, cognitive demand; (a) non-monotonicity, (b) non-repetitiveness, (c) complexity, (d) learning new things, (e) applying own ideas, and (f) solving unforeseen problems. Except for “applying own ideas”, which was a 5-point Likert scale, all components were rated using a dichotomous scale. Thus, the “applying own ideas” was set to 1 for “Always” or “Most of the time” and 0 for “Sometimes”, “Rarely”, or “Never”. The scores of these six components were combined to produce WCTV scores of 0–6 points (Cronbach’s alpha = 0.481 in this study). WCTV scores of 0–3 points were defined as low and scores of 4–6 as high. The KWCS also requested details of working hours. According to the Korean labor act, we defined 41–51 h per week as the standard and classified LW as 52–60 h (L1) or >60 h (L2). NW was defined as working more than 2 h between 10 p.m. and 5 a.m. In this case, the standard group was composed of individuals that did not work during the night over the previous month. NW was classified as 1–10 days per month (N1) and >10 days per month (N2). Occupations were classified as blue-collar or white-collar and according to the Korean Standard Classification of Occupations as; managers, professionals, clerks, service and sale workers (white-collar workers), and skilled agricultural, forestry and fishery workers, craft and related trades workers, plant, machine operators, and elementary occupations (blue-collar workers). About subjective health conditions, participants rated their overall subjective health conditions as one of “Very bad”, “Bad”, “Fair”, “Good”, and “Very good”. In this study, we classified them into “Bad”, “Fair”, and “Good”. Besides, sex, age, education level, number of employees, and monthly income were included in this study. Finally, we included four working time-related variables that might influence the effects of LW and NW on subjective well-being, that were; (a) working-time mismatch, (b) working-time variability, (c) shift work, and (d) working-time autonomy. Regarding working-time mismatch, we compared actual and desired working hours and classified them as “adequate”, “insufficient”, and “excessive”. For working-time autonomy, if scheduled work was fixed by the company or the worker could choose one of several schedules fixed by the company, the variable was set at “No”. On the other hand, if a worker could adapt his/her working hours within certain limits, or entirely determine the work schedule, the variable was set at “Yes”. The variability in working-time and shift work were expressed as yes or no.

### 2.3. Statistical Analysis

First, we conducted the Chi-square test and linear-by-linear association were used to identify trends of general characteristics for LW and NW. Second, the Chi-square test was used to determine whether the proportion of participants with LSW depended on LW or NW. When results were significant, we performed a pairwise comparison of proportions using Z-statistic. P-values were adjusted for multiple comparisons using Bonferroni’s correction method. Third, we conducted multiple logistic regression to examine the effects of LW and NW on subjective well-being and calculated Odds ratios (ORs) and 95% Confidence Intervals (CIs). Logistic regression analysis was performed on all study subjects and participants classified by WCTV and occupation. Fourth, we performed multiple logistic regression analysis adjusted for four variables that might influence the effects of LW and NW. Finally, we examined the difference in ORs of LW and NW for LSW from the model “after adjusted” for working time time-related variables to the model “not adjusted”, to investigate the effect sizes of variables and directions of changes. A positive value indicated that the variable increased the risks of LW or NW. A change was considered “substantial” when the ORs was changed by >10% or the statistical significance was changed. All statistical analyses were performed using IBM SPSS Statistics for Windows, version 25.0 (IBM Crop., Armonk, NY, USA).

## 3. Results

### 3.1. Distribution of Demographic Variables According to LW and NW

The characteristics of the study group and distributions according to LW and NW are shown in Table 1. All variables showed significant results by the Chi-square and trend tests. Men performed more LW and NW, and workers in large companies had shorter working hours and performed more NW. In this study, workers with low WCTV performed more LW and NW. Blue-collar workers performed more NW but worked fewer hours than white-collar workers.

### 3.2. LW and NW According to WCTV and Occupation

**Figure 1:**
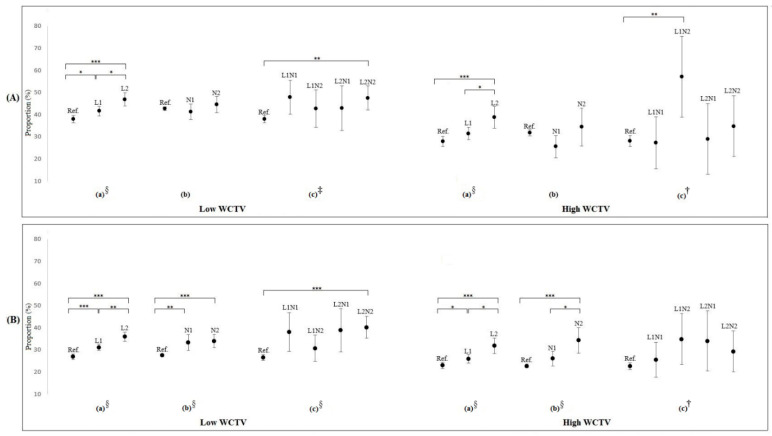
Proportions of those with low subjective well-being according to long working hours and night work, subdivided by work creativity and task variety (WCTV) and occupation (**A**) blue collar workers, (**B**) white collar workers). Note: L1 indicates 52–60 h per week, L2 indicates >60 h per week, N1 indicates night work for 1–10 days per month, and N2 indicates night work for >10 days per month (* *p* < 0.05, ** *p* < 0.01, *** *p* < 0.001 for pairwise comparison and ^†^ *p* < 0.05, ^‡^ *p* < 0.01, ^§^ *p* < 0.001 for chi-square test).

Figure 1 shows the proportion of workers with LSW according to LW and NW, subdivided by WCTV and occupation. A higher proportion of blue-collar workers had LSW than white-collar, and a lower proportion of workers with high WCTV exhibited LSW. By occupation, blue-collar workers did not show a difference in LSW for both N1 and N2, as compared with reference (workers with no NW). In contrast, white-collar workers showed a higher proportion of LSW when they get N2. Figure 1 shows different aspects according to WCTV. Unlike the low WCTV groups, L1 did not exhibit a difference as compared with reference (workers with standard working hours) in the blue-collar high WCTV groups. In the blue-collar high WCTV groups, only L2 showed a relatively high proportion of LSW. The synergistic effect of LW and NW was not clear in this figure. In the low WCTV groups, only L2N2 showed a higher proportion of LSW as compared with the reference group (workers with standard working hours and no NW). For blue-collar workers with high WCTV, only L1N2 showed a difference in LSW. No difference was found for white-collar workers with high WCTV group.

### 3.3. Results of Multiple Logistic Regression for LSW

Table 2 shows the results of multiple logistic regression for LSW divided by occupation and WCTV. Regression analysis of all study subjects showed that all variables except for sex were related to LSW. Increases in LSW proportions were observed when workers performed LW (OR 1.18 for L1, OR 1.30 for L2) or NW (OR 1.17 for N1, OR 1.14 for N2). For WCTV and occupation, high WCTV (OR 0.87) and white-collar work (OR 0.82) were associated with lower LSW proportions. Regression analysis performed by occupation and WCTV showed LW generally increased LSW as compared to standard hours. L2 increased LSW regardless of occupation and WCTV, but L1 had a significant effect only in the low WCTV group. Regarding short working hours, for blue-collar workers, working less than the standard working hours had a negative effect on subjective well-being (significant for those with low WCTV), but a positive effect was observed for white-collar (significant for those with high WCTV). NW also had different effects on LSW according to occupation and WCTV. N1 had no significant effect on subjective well-being in the high WCTV groups. In the high WCTV blue-collar group, no NW of any kind increased or decreased LSW, and in the high WCTV white-collar group, only N2 considerably increased the proportion with LSW (OR 1.49). In the case of low WCTV groups, only N1 increased LSW (OR 1.22 for blue-collar, OR 1.28 for white-collar).

Table 3 shows the results of multiple logistic regression for LSW after adjusting for working time-related variables. Model 1 showed results adjusted for working-time mismatch. Regardless of occupation and WCTV, insufficient working hours increased LSW. In the case of excessive work, results showed a tendency toward LSW increase; only the low WCTV group showed a significant association. Model 2 produced results adjusted for working-time variability, which increased LSW proportions in all groups. In model 3, the effect of the shift generally decreased LSW, and this was significant in low WCTV groups. Model 4 showed working-time autonomy increased LSW in blue-collar workers with low WCTV and white-collar workers with high WCTV.

**Figure 2:**
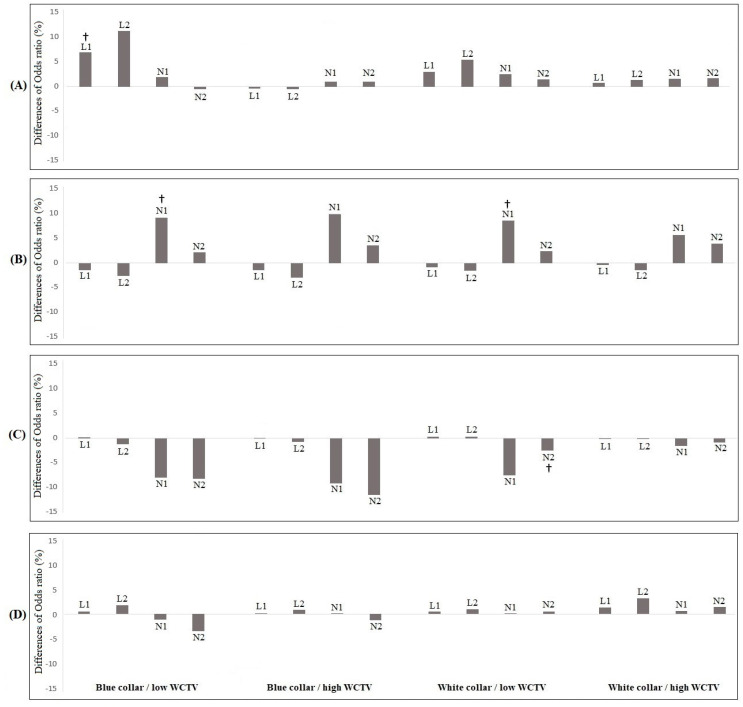
Differences in the odds ratios of long working hours and night work on low subjective well-being from a model adjusted for (**A**) working-time mismatch, (**B**) working-time variability, (**C**) shift work, and (**D**) working-time autonomy to the model not adjusted. L1 indicates 52–60 h per week, L2 indicates >60 h per week, N1 indicates night work for 1–10 days per month, and N2 indicates night work for >10 days per month. A change was considered “substantial” when the ORs changed by >10% or the statistical significance changed (†: A change in statistical significance, WCTV: work creativity and task variety). Odds ratio adjusted for all other confounders (sex, age, education level, numbers of employee, subjective health condition, and monthly income).

Figure 2 shows the OR differences for LW and NW from the model “after adjusted” for the variables shown in Table 3 to the model “not adjusted”. In the figure, a positive value means that the variable increased the risks of LW or NW. (A) and (B) show that the risk of LW and NW was generally reinforced, respectively, and (C) shows that the risk of NW was weakened. (A) shows that working-time mismatch influenced the risk of LW and that the risk of LW was more reinforced in low WCTV groups, a change in significance was only seen in L1 of blue-collar with low WCTV group. The influence of working-time variability is shown in (B). A change in the significance of NW was only observed in the low WCTV groups. (C) shows the influence of shift work on the effects of LW and NW. Similar to that shown in (B), OR differences were mainly observed for NW. However, unlike (B), shift work reduced the risk posed by NW, while variability in working times generally increased the risk of NW. Relatively little change in OR was caused by adjusting for working-time autonomy.

## 4. Discussion

**Figure 3:**
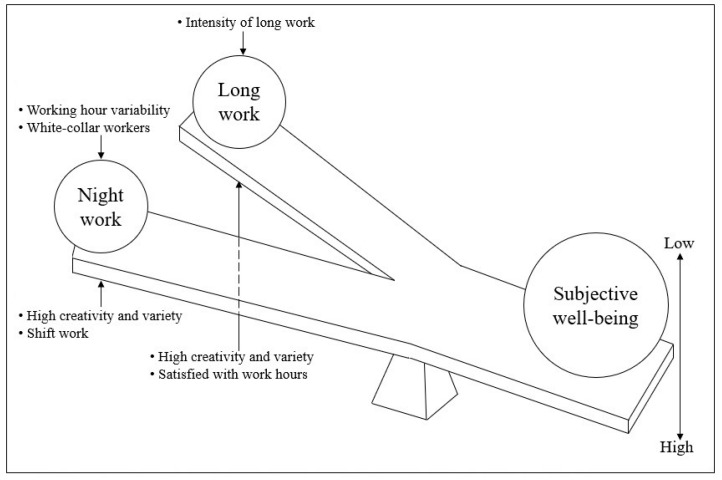
Theoretical framework of relationship between subjective well-being, long working hours, and night work.

The results of this study showed that LW and NW have negative effects on subjective well-being. Previous studies explained the negative effect of LW and NW on mental health by circadian rhythm disruption, sleep disturbance, lack of time to recover from work, and work-family conflicts [18,23,24,25]. Furthermore, the present study shows that creative and varied tasks have a positive effect on subjective well-being. Valcour explained that more complex tasks allowed workers autonomy and discretion, which promoted worker development such as in terms of skills and psychological resources [13]. The present study shows that working-time mismatch was found to be associated with the risks posed by LW, while variability of working time and shift work were associated with NW. However, working-time autonomy was less associated with LW or NW. This differs from a study conducted in the United States, which showed that control of work schedule enhanced work-life balance [26]. A previous study showed that the adoption rate of a flexible work system in Korea (12.5%) was considerably lower than that in the U.S. (81.0%) and Europe (66.0%) [27]. As a result, our observation could be attributed to the Korean labor culture in which the flexible work system was poorly utilized. Further study is needed to determine how the effects of autonomy with respect to working time depend on labor culture. We presented a theoretical framework based on the above results. On the left side, LW and NW act to decrease subjective well-being, and variables such as WCTV and occupation act to strengthen or weaken the effects. For example, high WCTV alleviated the risk of LW and NW and improved subjective well-being (Figure 3).

The synergistic effect of LW and NW was not demonstrated in this study, which contrasts with a prior study [28]. For blue-collar workers with high WCTV, a change in the LSW proportion was observed for L1N2 but not for L2N2. This could be interpreted as a result of a specificity of being blue-collar and being creative at the same time. In this study, desired working hours per week was longest in blue-collar workers with high WCTV. Furthermore, both enthusiasm at work and monthly income were higher than in the white-collar low WCTV group. A previous study showed that voluntary overtime work was less harmful than involuntary work, and rewards had a central role in the risk of overtime [29]. These findings suggest that the stress caused by simultaneous LW and NW might be relatively low in blue-collar workers with high WCTV.

### 4.1. LW and Subjective Well-Being

Although LW generally had a negative effect on subjective well-being, its effects depended on WCTV. L1 had a negative effect on subjective well-being in groups with a low WCTV, but had no significant effect for blue-collar workers with high WCTV (Figure 1). Moreover, the results of multiple logistic regression also showed that L1 did not affect LSW in groups with high WCTV, which suggested workers with high creativity work are less sensitive to LW. These results could be interpreted using model 1 in Table 3 and Figure 2A. Table 3 shows that the risk of LW was modified after adjusting for working-time mismatch, which concurs with a previous study [16]. Table 3 shows that subjective excessive work (when actual working hours were longer than wished) did not reduce subjective well-being in the high WCTV groups. Similarly, Figure 2A showed that the differences in the OR of LW after adjusting the working-time mismatch were relatively small in the high WCTV group. The above results suggested that individuals in the low WCTV groups were more sensitive to “working more than they wished,” indicating that even low-intensity LW could have an adverse effect on subjective well-being and that much of this adverse effect resulted from working-time mismatch.

### 4.2. NW and Subjective Well-Being

In this study, the effect of NW was affected by WCTV and occupation. In model 2 of Table 3, with the exception of the high WCTV white-collar group, NW did not have a significant effect on subjective well-being after adjusting for working-time variability. This result is attributed to a significant decrease in the OR of N1. In other words, it suggests that the risk of N1 could be due to unstable working hours rather than the fact that workers worked at night, as shown in Figure 2. Previous studies showed that working-time variability was an important cause of family conflict and caused workers to lose control of time schedules and personal plans [27,30]. The marked dependence of the risk of NW on working-time variability, also explains why N2 had a less detrimental effect on subjective well-being than N1 in Table 2, with the exception of high WCTV white-collar workers. Working more than 10 days on nights per month describes a normalized form of work, which had less harmful consequences than irregular NW. In fact, in this study, 45% of N2 workers worked at night almost every day of the month except weekends (>21 days per month) (data not shown).

When we adjusted for shift work, some interesting results were obtained. As shown in Figure 2, shift work resulted in a reduction in the LSW risk of NW, especially among blue-collar workers, suggesting that the reason why blue-collar workers were relatively insensitive to the risk of NW could be due to the effects of shift work. However, this result contrasts with those of previous studies, which reported shift work had negative effects on subjective well-being [31,32,33]. This study was able to infer the cause of these positive effects of shift work from the EWCS report. Workers in the health sector, one of the most representative areas for implementing shift in Europe, were most associated with working as a team (69%) [1]. Similarly, workers in electricity, gas, steam and water supply sectors, which accounted for most shift work in KWCS, also had the highest percentage of working as a team (48.5%). In addition, among night workers, shift workers (49.8%) were found to be much more team-based than non-shift workers (20.7%) in this study (Appendix A). For night workers, working as a team or alone might be an important factor of subjective well-being. In stressful situations, many studies have shown that social support from coworkers alleviates the negative effects of stress [34,35].

In addition, the unique characteristic of shift work could explain how the negative effects of NW were alleviated, given that the purpose of shift work often differs from that of other workers. For example, shift workers are often employed to maintain certain conditions (e.g., patient health or the freshness of goods) rather than to achieve performance-orientated goals. Therefore, shift workers work at speed to perform monotonous tasks [1], which is also shown by Appendix A. In the present study, these low WCTV-like characteristics were found to negatively affect subjective well-being. However, this is likely to have different effects on night workers, because, during NW, these monotonous task characteristics could alleviate the symptoms of worker exhaustion [36]. In fact, in Appendix A, the entire sample showed that shift workers (24.1%) reported higher levels of exhaustion than non-shift workers (21.4%). However, among night workers, the exhaustion level was lower in shift workers (22.4%) than in non-shift workers (24.1%). Summarizing, this study shows the above characteristics of shift work (team-based and low WCTV-like work) could alleviate the risk of NW on subjective well-being. Nonetheless, additional studies are required to determine the inherent characteristics of shift work.

### 4.3. Limitations

The first limitation of this study concerns the possibility of generalization. The results of this study are representative of Korean workers, but may not be representative of other nationalities. For example, our results concerning working-time autonomy conflicted with results obtained in a US study [26]. Furthermore, because labor cultures differ between countries, it may be difficult to apply our results equitably. Second, there is a problem of misclassification, as variables such as LW and NW were assessed by participant recall. In addition, the division of subjective well-being variables about cut-off values may have been an oversimplification, although it allowed us to use multiple logistic regression analysis to better understand the extents to which variables affected subjective well-being. In addition, previous studies have verified the validity of using 50 points as a cut-off value for poor well-being [22]. Third, researcher or self-response bias may have influenced data collection. However, the KWCS data collected during a national-scale survey did not focus on LW, NW, or subjective well-being. Thus, the risks of intentional and unintentional biases were relatively low. Fourth, we could not include variables such as marital status and self-efficacy, which reportedly have impacts on well-being [37]. Marital status could not be included in our study because the KWCS only contained information about family members living together and did not detail separated families or bereavements. The inclusion of such data may have strengthened or weakened relations identified [38,39]. Fifth, since responses to only one of the six questions were rated using a 5-point Likert scale in the process of constructing WCTV variable, information loss may have occurred when converting data to a dichotomous scale. In addition, WCTV had a relatively low Cronbach’s alpha value (0.481). This limitation was due to the lack of objective tools to measure WCTV in the data set. Consequently, additional research using validated measurement tools such as Work Design Questionnaire (WDQ) [40] and KEYS [41] is required. However, the composition of WCTV was similar to the decision latitude scale of the Job Content Questionnaire (JCQ) [42] and the Demand Control Support Questionnaire (DCSQ) [43], and the results of previous studies related to WCTV were similar to those obtained in the present study [44,45], which means that our WCTV measurements were largely valid. In addition, a prior study reported that the cut-off value for an acceptable and sufficient Cronbach’s alpha value is 0.45 [46]. Sixth, a change in statistical significance or OR of >10% were used as criteria of a “substantial” change. This was an operational definition rather than an absolute definition because it had no mathematical or statistical basis. Seventh, the statistical significance in this study might have been high, even if the effect of the variables was not heavy in practice, because the data we currently use had large samples. Finally, due to the cross-sectional nature of the study, we could not access the causalities of relationships. We suggest a longitudinal study be conducted to address these shortcomings. Nevertheless, this study is meaningful as it identifies the modulating effects of occupational characteristics and WCTV on relationships between subjective well-being, LW and NW.

## 5. Conclusions

Both LW and NW had negative effects on subjective well-being. High level of WCTV alleviated both the risk of LW and NW. White-collar workers were more vulnerable to the risk associated with NW as compared to blue-collar workers. Accordingly, it is necessary to comprehensively consider the occupation and task characteristics of workers performing LW or NW. In addition, working-time mismatch and variability increase the risk of LW and NW, respectively, and thus, workers’ desired working hours need to be reflected in working-time arrangements (especially in low WCTV workers), and NW needs to be adjusted to be as regular as possible. Performing shift work could alleviate the risks of NW. In countries where flexible work systems are not well utilized, as in Korea, working-time autonomy might not be associated with the risk of LW or NW. Further studies on the modifying effects of working time-related variables on LW and NW are needed.

## Figures and Tables

**Table 1:** Distributions of demographic variables according to working hours per week and night work.

Variables		n	%	Working Hours per Week				Chi-Square Test (Trend Test)	Night Work per Month			Chi-Square Test (Trend Test)
				<41	41–51	52–60	>60		0 Day	1–10 Days	Over 10 Days	
				n (%)	n (%)	n (%)	n (%)		n (%)	n (%)	n (%)	
Sex	Male	23,707	47.2	10,483 (44.4)	6653 (28.1)	4289 (18.1)	2210 (9.4)	*** (***)	20,660 (87.1)	1673 (7.1)	1374 (5.8)	*** (***)
	Female	26,498	52.8	13,772 (52.1)	6340 (24.0)	4458 (16.9)	1852 (7.0)		25,004 (94.4)	761 (2.9)	733 (2.8)	
Age	<39	12,954	25.8	6967 (53.9)	3580 (27.7)	1822 (14.1)	555 (4.3)	*** (***)	11,754 (90.7)	769 (5.9)	431 (3.3)	*** (*)
	40–49	11,795	23.5	5620 (47.8)	3269 (27.8)	2110 (17.9)	766 (6.5)		10,750 (91.1)	604 (5.1)	441 (3.7)	
	50–59	13,250	26.4	5322 (40.3)	3608 (27.3)	2883 (21.8)	1399 (10.6)		12,001 (90.6)	608 (4.6)	641 (4.8)	
	> 59	12,206	24.3	6346 (52.2)	2536 (20.9)	1932 (15.9)	1342 (11.0)		11,159 (91.4)	453 (3.7)	594 (4.9)	
Educational level	Under high school	9751	19.4	5440 (56.0)	1936 (19.9)	1357 (14.0)	978 (10.1)	*** (***)	9112 (93.4)	277 (2.8)	362 (3.7)	*** (**)
	High school	19,156	38.2	7102 (37.2)	5276 (27.6)	4573 (23.9)	2143 (11.2)		17,004 (88.8)	997 (5.2)	1155 (6.0)	
	Bachelor’s degree	20,600	41.1	11,217 (54.6)	5658 (27.5)	2762 (13.4)	921 (4.5)		18,909 (91.8)	1113 (5.4)	578 (2.8)	
	Masters or more	646	1.3	469 (73.1)	112 (17.4)	45 (7.0)	16 (2.5)		591 (91.5)	43 (6.7)	12 (1.9)	
Numbers of employee	1	12,863	25.8	4258 (33.2)	3071 (24.0)	3748 (29.2)	1740 (13.6)	*** (***)	11,867 (92.3)	427 (3.3)	569 (4.4)	*** (***)
	2–9	19,909	39.9	8689 (43.8)	5595 (28.2)	3732 (18.8)	1829 (9.2)		18,256 (91.7)	659 (3.3)	994 (5.0)	
	10–49	10,255	20.5	6437 (62.9)	2698 (26.4)	791 (7.7)	311 (3.0)		9325 (90.9)	605 (5.9)	325 (3.2)	
	50–249	4465	8.9	2976 (66.7)	1049 (23.5)	309 (6.9)	126 (2.8)		3952 (88.5)	373 (8.4)	140 (3.1)	
	Over 249	2426	4.9	1694 (69.9)	529 (21.8)	154 (6.4)	45 (1.9)		2004 (82.6)	357 (14.7)	65 (2.7)	
Subjective health condition	Good	33,058	65.9	16,263 (49.3)	8706 (26.4)	5714 (17.3)	2292 (7.0)	*** (***)	30,169 (91.3)	1643 (5.0)	1246 (3.8)	*** (***)
	Fair	14,471	28.8	6463 (44.8)	3822 (26.5)	2671 (18.5)	1470 (10.2)		13,015 (89.9)	710 (4.9)	746 (5.2)	
	Bad	2669	5.3	1527 (57.6)	462 (17.4)	361 (13.6)	300 (11.3)		2474 (92.7)	81 (3.0)	114 (4.3)	
Monthly income	< $1501	13,688	29.8	8647 (63.3)	2691 (19.7)	1446 (10.6)	887 (6.5)	*** (***)	12,771 (93.3)	393 (2.9)	524 (3.8)	*** (***)
	$1501–$2900	21,622	47.1	8541 (39.6)	6509 (30.2)	4598 (21.3)	1938 (9.0)		19,576 (90.5)	1111 (5.1)	935 (4.3)	
	$2901–$3800	6191	13.5	2765 (44.7)	1741 (28.1)	1222 (19.8)	458 (7.4)		5512 (89.0)	431 (7.0)	248 (4.0)	
	> $3800	4426	9.6	2002 (45.3)	1144 (25.9)	841 (19.0)	433 (9.8)		3846 (86.9)	353 (8.0)	227 (5.1)	
Work creativity and task variety	Low	33,210	66.4	15,887 (48.0)	8487 (25.6)	5722 (17.3)	3003 (9.1)	*** (***)	30,040 (90.5)	1453 (4.4)	1717 (5.2)	*** (***)
	High	16,839	33.6	8293 (49.3)	4474 (26.6)	2999 (17.8)	1048 (6.2)		15,486 (92.0)	969 (5.8)	384 (2.3)	
Occupation	Blue collar	18,987	37.9	9461 (50.0)	5008 (26.5)	3007 (15.9)	1439 (7.6)	*** (***)	17,098 (90.1)	1048 (5.5)	841 (4.4)	*** (***)
	White collar	31,125	62.1	14,728 (47.4)	7963 (25.6)	5736 (18.5)	2623 (8.4)		28,499 (91.6)	1360 (4.4)	1266 (4.1)	
Subjective well-being	High	34,384	68.6	16,727 (48.7)	9194 (26.8)	5904 (17.2)	2500 (7.3)	*** (***)	31,447 (91.5)	1625 (4.7)	1312 (3.8)	*** (***)
	Low	15,770	31.4	7510 (47.8)	3790 (24.1)	2836 (18.1)	1559 (9.9)		14,170 (89.9)	806 (5.1)	794 (5.0)	

* *p* < 0.05, ** *p* < 0.01, *** *p* < 0.001.

**Table 2:** Results of multiple logistic regression related to low subjective well-being according to work creativity and task variety, and occupation. Odds ratio (95% Confidence Interval).

Covariates		All (n = 45,467)	Blue Collar Worker		White Collar Worker	
			Work Creativity and Task Variety			
			Low (n = 12,761)	High (n = 4150)	Low (n = 17,268)	High (n = 11,288)
Sex	Male	Ref.	Ref.	Ref.	Ref.	Ref.
	Female	0.96 (0.91–1.01)	0.89 (0.82–0.97) **	0.78 (0.65–0.94) *	1.05 (0.97–1.14)	0.96 (0.87–1.06)
Age	<39	Ref.	Ref.	Ref.	Ref.	Ref.
	40–49	1.17 (1.10–1.25) ***	1.20 (1.03–1.41) *	0.94 (0.74–1.18)	1.15 (1.05–1.27) **	1.22 (1.09–1.37) ***
	50–59	1.12 (1.04–1.19) **	1.03 (0.89–1.20)	0.97 (0.77–1.22)	1.21 (1.09–1.34) ***	1.04 (0.91–1.19)
	>59	1.10 (1.02–1.20) *	0.99 (0.85–1.16)	0.95 (0.73–1.24)	1.25 (1.09–1.43) **	0.90 (0.73–1.11)
Educational level	Under high school	Ref.	Ref.	Ref.	Ref.	Ref.
	High school	0.73 (0.68–0.79) ***	0.75 (0.68–0.83) ***	0.98 (0.79–1.22)	0.70 (0.61–0.80) ***	0.64 (0.49–0.84) **
	Bachelor’s degree	0.66 (0.60–0.72) ***	0.65 (0.56–0.76) ***	0.93 (0.71–1.20)	0.61 (0.53–0.71) ***	0.59 (0.44–0.78) ***
	Masters or more	0.70 (0.56–0.88) **	0.35 (0.10–1.27)	2.21 (0.60–8.13)	0.85 (0.57–1.27)	0.55 (0.38–0.80) **
Numbers of employee	1	Ref.	Ref.	Ref.	Ref.	Ref.
	2–9	1.01 (0.96–1.07)	1.06 (0.97–1.16)	0.93 (0.78–1.10)	1.04 (0.95–1.14)	1.03 (0.90–1.18)
	10–49	1.04 (0.97–1.11)	0.93 (0.83–1.04)	0.73 (0.58–0.91) **	1.24 (1.11–1.39) ***	1.14 (0.98–1.34)
	50–249	1.04 (0.95–1.13)	0.96 (0.82–1.12)	0.79 (0.59–1.06)	1.07 (0.92–1.26)	1.24 (1.03–1.48) *
	Over 249	1.16 (1.04–1.30) **	0.98 (0.79–1.21)	0.86 (0.61–1.20)	1.09 (0.87–1.37)	1.56 (1.27–1.92) ***
Subjective health condition	Good	Ref.	Ref.	Ref.	Ref.	Ref.
	Fair	2.19 (2.09–2.29) ***	2.23 (2.06–2.42) ***	2.60 (2.23–3.03) ***	2.04 (1.89–2.20) ***	2.27 (2.04–2.53) ***
	Bad	4.54 (4.12–5.01) ***	4.56 (3.99–5.21) ***	6.75 (4.88–9.33) ***	3.60 (2.95–4.40) ***	5.19 (3.78–7.11) ***
Monthly income	< $1501	Ref.	Ref.	Ref.	Ref.	Ref.
	$1501–$2900	0.83 (0.79–0.88) ***	0.80 (0.73–0.88) ***	0.76 (0.61–0.93) **	0.85 (0.78–0.93) ***	0.87 (0.75–1.01)
	$2901–$3800	0.81 (0.75–0.88) ***	0.71 (0.60–0.84) ***	0.79 (0.61–1.02)	0.86 (0.75–0.98) *	0.84 (0.70–1.00) *
	>$3800	0.69 (0.63–0.76) ***	0.63 (0.51–0.79) ***	0.62 (0.46–0.84) **	0.76 (0.65–0.89) ***	0.69 (0.57–0.84) ***
Working hours per week	41–51	Ref.	Ref.	Ref.	Ref.	Ref.
	<41	1.00 (0.94–1.05)	1.10 (1.00–1.21) *	1.16 (0.97–1.38)	0.92 (0.85–1.01)	0.85 (0.76–0.96) **
	52–60	1.18 (1.10–1.26) ***	1.16 (1.03–1.32) *	1.15 (0.95–1.39)	1.23 (1.11–1.36) ***	1.15 (0.99–1.33)
	>60	1.30 (1.20–1.42) ***	1.40 (1.19–1.64) ***	1.43 (1.09–1.88) **	1.26 (1.11–1.44) ***	1.28 (1.04–1.57) *
Night work per months	0 day	Ref.	Ref.	Ref.	Ref.	Ref.
	1–10 days	1.17 (1.06–1.29) **	1.22 (1.04–1.44) *	0.95 (0.70–1.28)	1.28 (1.07–1.53) **	1.09 (0.90–1.32)
	Over 10 days	1.14 (1.02–1.26) *	1.10 (0.92–1.32)	0.93 (0.59–1.45)	1.14 (0.98–1.33)	1.49 (1.12–1.98) **
Work creativity and task variety	Low	Ref.				
	High	0.87 (0.83–0.92) ***				
Occupation	Blue collar	Ref.				
	White collar	0.82 (0.78–0.86) ***				
R^2		0.117	0.121	0.118	0.075	0.060

* *p* < 0.05, ** *p* < 0.01, *** *p* < 0.001. $ means dollar.

**Table 3:** Results of multiple logistic regression related to low subjective well-being according to work creativity and task variety and occupation after adjusting for working-time mismatch, variability, shift work, and autonomy. Odds ratio (95% Confidence Interval).

Covariates			Blue Collar Worker		White Collar Worker	
			Work Creativity and Task Variety			
			Low	High	Low	High
Model 1	Working hours per week	41–51	Ref.	Ref.	Ref.	Ref.
		<41	1.21 (1.10–1.34) ***	1.13 (0.94–1.36)	0.96 (0.87–1.05)	0.87 (0.77–0.98) *
		52–60	1.08 (0.95–1.22)	1.15 (0.95–1.40)	1.19 (1.07–1.32) ***	1.14 (0.98–1.32)
		>60	1.24 (1.05–1.46) **	1.44 (1.09–1.89) **	1.19 (1.05–1.36) **	1.26 (1.02–1.55) *
	Night work per months	0 day	Ref.	Ref.	Ref.	Ref.
		1–10 days	1.20 (1.01–1.41) *	0.94 (0.70–1.27)	1.25 (1.04–1.49) *	1.07 (0.88–1.30)
		Over 10 days	1.11 (0.93–1.33)	0.92 (0.59–1.44)	1.12 (0.96–1.31)	1.47 (1.10–1.95) **
	Working-time mismatch	Adequate	Ref.	Ref.	Ref.	Ref.
		Insufficient	1.26 (1.13–1.41) ***	1.51 (1.16–1.95) **	1.36 (1.20–1.54) ***	1.41 (1.17–1.71) ***
		Excessive	1.58 (1.44–1.74) ***	1.10 (0.93–1.30)	1.28 (1.18–1.40) ***	1.12 (0.99–1.26)
Model 2	Working hours per week	41–51	Ref.	Ref.	Ref.	Ref.
		<41	1.08 (0.99–1.19)	1.16 (0.97–1.38)	0.93 (0.85–1.01)	0.86 (0.77–0.96) **
		52–60	1.18 (1.04–1.34) **	1.16 (0.96–1.41)	1.24 (1.12–1.37) ***	1.15 (0.99–1.34)
		>60	1.44 (1.22–1.69) ***	1.47 (1.12–1.93) **	1.28 (1.13–1.46) ***	1.29 (1.05–1.59) *
	Night work per months	0 day	Ref.	Ref.	Ref.	Ref.
		1–10 days	1.11 (0.94–1.31)	0.85 (0.63–1.16)	1.17 (0.98–1.40)	1.03 (0.84–1.25)
		Over 10 days	1.08 (0.90–1.29)	0.89 (0.57–1.40)	1.11 (0.95–1.30)	1.43 (1.08–1.91) *
	Working-time variability	No	Ref.	Ref.	Ref.	Ref.
		Yes	1.28 (1.17–1.39) ***	1.29 (1.10–1.51) **	1.30 (1.18–1.43) ***	1.17 (1.03–1.32) *
Model 3	Working hours per week	41–51	Ref.	Ref.	Ref.	Ref.
		<41	1.10 (1.00–1.21) *	1.16 (0.97–1.38)	0.93 (0.85–1.01)	0.86 (0.76–0.96) **
		52–60	1.16 (1.02–1.31) *	1.15 (0.95–1.39)	1.22 (1.11–1.36) ***	1.15 (0.99–1.33)
		>60	1.42 (1.21–1.66) ***	1.44 (1.10–1.89) **	1.26 (1.11–1.43) ***	1.28 (1.04–1.57) *
	Night work per months	0 day	Ref.	Ref.	Ref.	Ref.
		1–10 days	1.32 (1.10–1.58) **	1.04 (0.74–1.44)	1.38 (1.15–1.65) ***	1.11 (0.90–1.36)
		Over 10 days	1.19 (0.98–1.45)	1.03 (0.64–1.67)	1.17 (1.00–1.37) *	1.50 (1.13–2.00) **
	Shift work	No	Ref.	Ref.	Ref.	Ref.
		Yes	0.85 (0.73–0.99) *	0.80 (0.57–1.14)	0.83 (0.73–0.94) **	0.95 (0.77–1.18)
Model 4	Working hours per week	41–51	Ref.	Ref.	Ref.	Ref.
		<41	1.11 (1.01–1.22) *	1.16 (0.98–1.39)	0.93 (0.85–1.01)	0.86 (0.77–0.96) **
		52–60	1.16 (1.02–1.31) *	1.15 (0.95–1.39)	1.22 (1.10–1.36) ***	1.13 (0.98–1.31)
		>60	1.38 (1.17–1.61) ***	1.42 (1.08–1.86) *	1.25 (1.10–1.42) ***	1.23 (1.00–1.52) *
	Night work per months	0 day	Ref.	Ref.	Ref.	Ref.
		1–10 days	1.24 (1.05–1.46) *	0.95 (0.70–1.28)	1.28 (1.07–1.52) **	1.08 (0.89–1.31)
		Over 10 days	1.14 (0.96–1.37)	0.94 (0.60–1.47)	1.14 (0.97–1.33)	1.47 (1.11–1.95) **
	Working-time autonomy	No	Ref.	Ref.	Ref.	Ref.
		Yes	1.27 (1.16–1.40) ***	1.13 (0.94–1.36)	1.06 (0.96–1.16)	1.21 (1.06–1.37) **

* *p* < 0.05, ** *p* < 0.01, *** *p* < 0.001. Odds ratio adjusted for all other confounders (sex, age, education level, numbers of employee, subjective health condition, monthly income).

## Data Availability

The raw KWCS data fo published paper used in this study is available from reference [20]. The data of report is available from the Occupational Safety and Health Research Institute (OSHIR). However, this data available with the approbal of the OSHIR.

## Appendix A

**Table A1:** Distribution of variables according to shift work among all study subjects and night workers.

Variables		Entire Sample		*p*-Value	Workers with Night Work		*p*-Value
		No Shift	Shift		No Shift	Shift	
		n (%)	n (%)		n (%)	n (%)	
Work as team or group ^†^	Yes	10,146 (22.1)	1792 (42.3)	***	545 (20.7)	946 (49.8)	***
	No	35,668 (77.9)	2445 (57.7)		2084 (79.3)	952 (50.2)	
Work creativity and task variety	High	15,855 (34.6)	980 (23.2)	***	851 (32.3)	502 (26.6)	***
	Low	29,954 (65.4)	3250 (76.8)		1782 (67.7)	1388 (73.4)	
Exhaustion after work ^‡^	High	9811 (21.4)	1023 (24.1)	***	688 (26.1)	425 (22.4)	**
	Low	36,026 (78.6)	3218 (75.9)		1951 (73.9)	1472 (77.6)	

*p*-values were determined using the Chi-square test. ^†^ The question used to address “work as a team or group” was “Do you work in a group or team that has common tasks and can plan its work?”. ^‡^ The question used to address “exhaustion after work” was “ How often do you feel exhausted at the end of the working day?”. Workers who answered “always” or “most of the time” were considered at ‘high’ risk of exhaustion after work, and workers who answered “sometimes”, “rarely”, “never” were considered at ‘low’ risk. ** p < 0.01, *** *p* < 0.001.
